# Chlorophyll decomposition is accelerated in banana leaves after the long-term magnesium deficiency according to transcriptome analysis

**DOI:** 10.1371/journal.pone.0270610

**Published:** 2022-06-24

**Authors:** Baolin Kan, Yong Yang, Pengmeng Du, Xinping Li, Wenjie Lai, Haiyan Hu

**Affiliations:** Hainan Key Laboratory for Sustainable Utilization of Tropical Bioresources, College of Tropical Crops, Hainan University, HaiKou, China; Selcuk University, TURKEY

## Abstract

Magnesium (Mg) is an essential macronutrient for plant growth and development. Physiological and transcriptome analyses were conducted to elucidate the adaptive mechanisms to long-term Mg deficiency (MD) in banana seedlings at the 6-leaf stage. Banana seedlings were irrigated with a Mg-free nutrient solution for 42 days, and a mock control was treated with an optimum Mg supply. Leaf edge chlorosis was observed on the 9^th^ leaf, which gradually turned yellow from the edge to the interior region. Accordingly, the total chlorophyll content was reduced by 47.1%, 47.4%, and 53.8% in the interior, center and edge regions, respectively, and the net photosynthetic rate was significantly decreased in the 9^th^ leaf. Transcriptome analysis revealed that MD induced 9,314, 7,425 and 5,716 differentially expressed genes (DEGs) in the interior, center and edge regions, respectively. Of these, the chlorophyll metabolism pathway was preferentially enriched according to Kyoto Encyclopedia of Genes and Genomes (KEGG) analysis. The expression levels of the five candidate genes in leaves were consistent with what is expected during chlorophyll metabolism. Our results suggest that changes in the expression of genes related to chlorophyll synthesis and decomposition result in the yellowing of banana seedling leaves, and these results are helpful for understanding the banana response mechanism to long-term MD.

## Introduction

Crops absorb magnesium ions (Mg^2+^) from the soil mainly through their roots, which are usually affected by various factors, such as soil texture, cation exchangeable capacity and climatic factors [1]. The unique chemical property of Mg^2+^ results in a bond with negatively charged root cell walls, promoting ion exchange with the soil [2]. However, soil saturated with cations would lead to Mg deficiency (MD), particularly in acidic soils in high rainfall areas [3]. MD has a major negative consequence on crop production, leading to a detrimental effect on yields and poor food and feed quality [4]. Thus, maintaining Mg contents for agricultural production is very important. In plants, approximately 5–35% of Mg is detected in chloroplasts, and Mg is the central element of the tetrapyrrole ring in chlorophyll [5, 6]. Mg usually accounts for ~0.15%-0.35% of the dry composition of vegetative organs but reaches ~6%-20% in chlorophyll [7]. MD has become a serious problem as a result of heavy rainfall and the inappropriate use of nitrogen, phosphorus, and potassium fertilizers [8, 9].

A substantial pool of total cellular Mg is required to synthesize chlorophyll in photosynthetic tissues, while the rest is used for ribosome bridging during translation and for chelation with nucleotides, nucleic acids and other phosphate-containing compounds [10]. As the central atom of chlorophyll, Mg is essential for photosynthesis in green plants. Chlorophyll acts in pigment-protein complexes to capture light and transfer the electrons in PSI and PSII [11, 12]. The first step for chlorophyll synthesis is the insertion of Mg^2+^ into protoporphyrin IX catalyzed by Mg-chelatase (MgCh) [13]. Mg-chelatase is a key enzyme at the branch point in the synthetic pathway of heme and chlorophyll, which is composed of the *ChlI*, *ChlH*, and *ChlD* submits in plants [14–16]. A series of genes encoding the enzymes involved in the Mg branch of the chlorophyll biosynthesis pathway, *viz*., CHLH, CHL1, CHLD, CHLM, CRD1, DVR, POR A/B/C and CHLG, have been identified using *in vitro* and *in vivo* approaches [17].

The typical chlorosis symptom of MD appears in the leaf intervein and usually appears first on the lower and older leaves owing to the mobile nature of Mg [18, 19]. At the early stage of MD in plants, photoassimilates accumulate in source leaves before photosynthesis is suppressed, resulting in an excessive accumulation of carbohydrates and enhanced production of reactive oxygen species (ROS) [6]. Later, excessive sucrose regulates the chlorophyll A/B binding protein 2 (*CAB2*) gene in a feedback mode, leading to a decreased chlorophyll concentration [20]. In *Arabidopsis*, *AtSGR1* and *AtSGR2*, which encode the enzyme Mg-dechelatase, are responsible for the breakdown of chlorophyll [21]. Under MD conditions, the expression of *OsSGR* is negatively regulated by ROS, affecting chlorophyll degradation in rice leaves [22]. miRNAs are also involved in plant MD tolerance [23]. As a result, MD affects dry matter production and carbohydrate partitioning in sugar bean, *Arabidopsis*, barley and *Citrus sinensis* [20, 24–26].

Banana is one of the world’s major food crops in the tropical and subtropical regions, where soil acidification has been a serious growing problem [27, 28]. As a result, MD has been a major risk factor for banana production. The impact of MD on the allocation of carbohydrates to different organs of the banana plant has been reported [29]. In contrast to model species such as *Arabidopsis*, rice, maize and soybean, the molecular mechanism of banana leaf chlorosis is unclear [21, 22, 30, 31]. Therefore, in the present study, an attempt was made to decipher the molecular mechanism of MD-induced banana leaf chlorosis through physiological and transcriptomic characterizations of banana (BaXi Jiao, *Musa acuminata*, AAA, cultivar Cavendish) seedling leaves exposed to long-term MD.

## Materials and methods

### Plant material and growth conditions

Banana (BaXi Jiao, *M*. *acuminata*, AAA, cultivar Cavendish) seedlings were purchased from the Danzhou Rapid Propagation of Banana Breeding Base (Hainan, China), cultivated in potting soil (Pindstrup, Denmark) until the 6-leaf stage and then used for MD treatment.

For MD treatment, all seedlings were transplanted to sand after rinsing the roots with double distilled water and then seedlings of uniform growth were randomly divided into two groups, *viz*., the MD and mock control groups. The modified Hoagland’s nutrient solution contained 4 mM Ca(NO_3_)_2_·4H_2_O, 2 mM NH_4_H_2_PO_4_, 4 mM KCl, 60 μM Fe-EDTA, 25 μM H_3_BO_3_, 2 μM MnSO_4_·H_2_O, 2 μM ZnSO_4_·7H_2_O, 0.5 μM CuSO_4_·5H_2_O, and 0.05 μM H_2_MoO_4_. For the mock control group, normal Hoagland’s nutrient solution containing 1 mM MgSO_4_·7H_2_O and 6 mM KNO_3_ was used, while 1 mM K_2_SO_4_, 4 mM KNO_3_ and 1 mM NH_4_NO_3_ were used to maintain the same K, S and N supply in the MD group. The two kinds of Hoagland’s nutrient solutions were supplied every 2 days. For each group, at least 15 plantlets were used, and 3 biological replicates were set. All seedlings were grown in the greenhouse of Hainan University. After 42 days of treatment, the 9^th^ leaves and the roots were collected, immediately frozen in liquid nitrogen and then stored at -80°C for RNA extraction. The 9^th^-leaf samples were divided into the edge (LM_E), center (LM_C) and interior (LM_I) regions.

### Photosynthesis and chlorophyll content measurement

After 42 days of treatment, photosynthetic parameters were measured in the 9^th^ leaf of the banana seedlings at 9:00–11:00 a.m. on a sunny day. All measurements were carried out using a CIRAS-3 portable photosynthesis system (PP Systems, USA). The photosynthetic photon flux density provided by a red/blue LED light source was amounted to 1200 μmol m^-2^s^-1^, the ambient CO_2_ concentration was adjusted to 390 μmol mol^-1^ by CO_2_ injection and the leaf temperature was maintained at 27°C.

The relative chlorophyll content of each leaf was measured using a chlorophyll meter (SPAD-502 Plus; Konica Minolta), and the SPAD value of the 9^th^ leaf in each plant was recorded from 31 to 60 days after treatment. The measurements were taken from at least three biological replicates per treatment, and the values were averaged.

### Biomass and Mg content analysis in leaves after long-term MD

After harvest, the plants were washed with deionized water to remove any residual ions. The roots, stems and leaves were heated at 105°C for 15–20 min, and then dried at 80°C until they reached a constant weight. The dry weight was measured and recorded.

For Mg content measurement, leaves were dried at 60°C for 3 days and then digested with nitric acid using a microwave digestion system (MILESTONE Ethos UP). After dilution in deionized water, the metal content in the samples was determined by ICP-MS (Agilent 7000 series).

### Transcriptome library construction and analysis of differentially expressed genes (DEGs)

Fresh samples were ground into powder in liquid nitrogen, and total RNA (at least 1 μg) was extracted from LM_I, LM_C, LM_E, and control samples using the RNA-prep Pure Plant Plus Kit (#DP441, TIANGEN, Beijing, China) according to the manufacturer’s instructions. After pre-processing and rRNA removal, a cDNA library was constructed using the Illumina NovaSeq 6000 platform (Majorbio Biology Company, Shanghai, China).

Clean reads were mapped based on the *Musa acuminate* genome by using the HISAT2 program, and the read count for each gene was obtained from mapping results [32]. Gene expression levels were estimated using the RSEM program [33]. DEGs between the two groups (MD and Mg sufficiency) were analyzed using the DESeq2 R package (1.24.0). Genes with an adjusted P value < 0.05 and |log2FC| ≥ 1 filtering condition following DESeq2 analysis were considered to be differentially expressed between groups. Gene Ontology (GO) enrichment analysis of the DEGs was conducted using GOATOOLS to determine over- and underrepresented terms [34]. KOBAS was used to test the significance of the enriched DEGs in special Kyoto Encyclopedia of Genes and Genomes (KEGG) pathways [35].

### Real-time quantitative polymerase chain reaction (qRT-PCR)

Five candidate genes related to chlorophyll metabolism were selected, and the primers were designed by Premier Primer 5.0 software (Premier Biosoft International, Palo Alto, CA, USA). The primer sequences and reference gene are listed in a supplemental table (S1 Table). cDNA synthesis was performed with a HiScript II 1st Strand cDNA Synthesis Kit (+gDNA wiper) (Vazyme, R212-01). Gene expression levels were analyzed by qRT-PCR on an ABI Q7 (Applied Biosystems, USA) using the ChamQ Universal SYBR qPCR Master Mix (Vazyme, Q711–02). The reaction mixtures, with a final volume of 5 μL, included 2× SYBR Mix, cDNA (1 μL), 0.2 μL each of forward and reverse primers, and PCR-grade water (3.6 μL). qPCR was performed under the following conditions: 95°C for 5 min, followed by 40 cycles at 95°C for 30s, 55°C for 30s, and 72°C for 40s. The qPCR was repeated three times for each gene in each sample, and experiments were performed in three biological replicates. The expression levels were calculated using the previous method [36]. The *MaRPS2* gene was used as an internal control.

## Results

### Long-term MD affected the growth of banana

**Fig 1:**
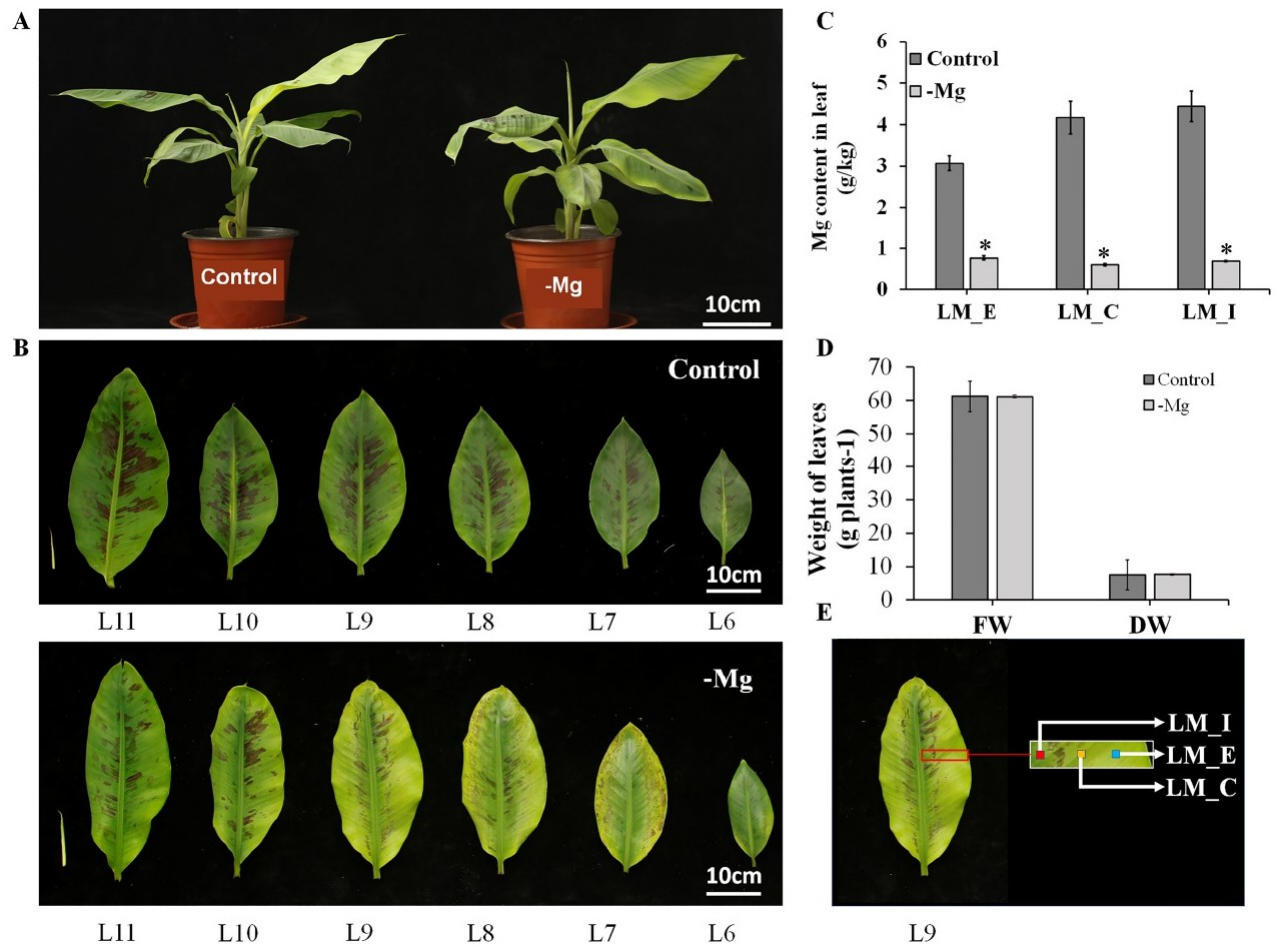
Phenotypes and Mg contents of banana (*Musa acuminata*) leaves under long-term MD. **(A)** The phenotype of banana seedlings after MD for 42 days. For the control groups, 1 mM Mg^2+^ Hoagland nutrient solutions were used, and 0 mM Mg^2+^ solutions were used for the long-term MD treatment (-Mg). Scale bars = 10 cm. **(B)** The phenotype of the 7^th^– 11^th^ banana seedling leaves between the control and the MD treatment. Scale bars = 10 cm. **(C)** Mg content in different parts of the 9^th^ leaves (L9) (*n* = 3). **(D)** Fresh weight and dry weight of 9^th^ leaf. FW: fresh weight, DW: dry weight. Values represent the mean ± standard error (SE) (*n* = 3). **(E)** The different parts of the 9^th^ leaf were collected for RNA-seq. LM_I represents leaf interior, LM_E represents leaf edge and LM_C represents leaf center. Data analysis was performed by two-tailed *t test*, * means significantly different.

Mg is essential to plant growth and development, especially for photosynthesis. To investigate the physiological changes in banana leaves in response to long-term MD stress, banana (BaXi Jiao, *M*. *acuminata*, AAA, cultivar Cavendish) seedlings at the 6-leaf-stage were planted in quartz sand without a Mg supply for 42 days (Fig 1A). The 9^th^ leaf (L9) was visible etiolated and showed symptoms of chlorosis (Fig 1A and 1B), which gradually decreased in decreased in degree from the edge to the inside regions (Fig 1B).

Compared to the control group, the Mg content of L9 decreased significantly (*p* < 0.01) under long-term MD in the LM_I, LM_C and LM_E regions (Fig 1C and 1E). Moreover, the Mg content gradually increased from the 8^th^ leaf (L8) to the 11^th^ leaf (L11) under the MD treatment, but showed the opposite trend in the control group (S1 Fig). However, MD stress had no significant (*p* = 0.99, 0.79) effect on the fresh and dried biomass of the 9^th^ leaf (Fig 1D). In conclusion, long-term MD led to chlorosis symptoms and lower Mg contents in banana leaves.

### Chlorophyll content and photosynthesis rate under long-term MD

**Fig 2:**
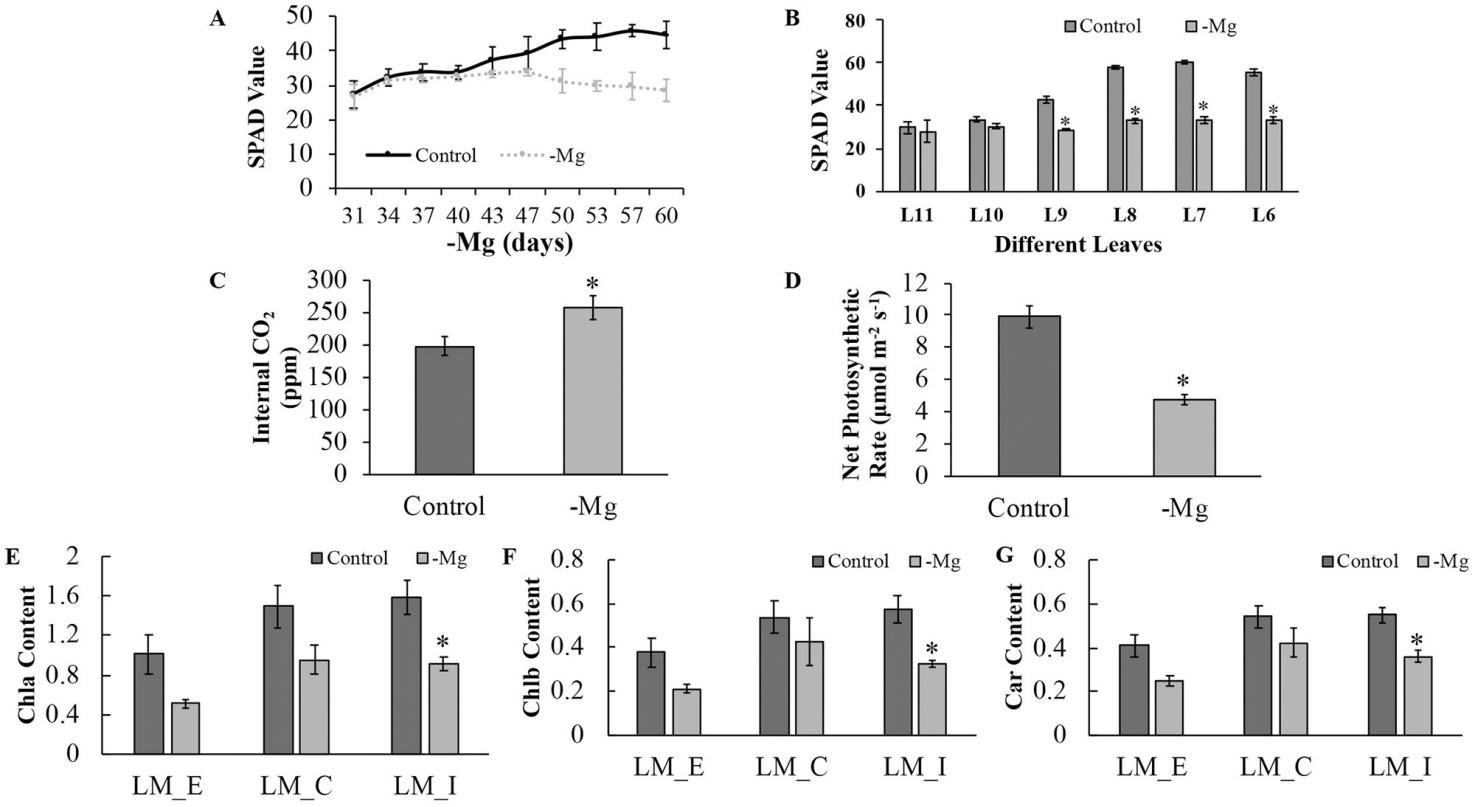
Physiological responses to MD stress among banana leaves. **(A)** SPAD values of L9 recorded from 31 to 60 days in the control and MD treatments (*n* = 5). (**B)** SPAD value variation from L6 to L11 on the 42nd day in the control and MD treatments (*n* = 24). **(C)** Internal CO_2_ concentrations of the MD treatment in L9 (*n* = 10). **(D)** Net photosynthetic rate (Pn) of the MD treatment in L9 (*n* = 10). **(E-G)** Chlb, Chla and Car contents in different regions of L9 (*n* = 3). Chla: chlorophyll a; Chlb: chlorophyll b; Car: carotenoid. Data analysis was performed by two-tailed *t test*, * means significantly different.

To explore the physiological process of banana leaves in response to long-term MD, the relative chlorophyll concentration (SPAD) was measured. The SPAD value of L9 showed a significant (*p* < 0.01) decrease after 60 days of MD treatment, with the differences starting at 40 days (Fig 2A). Moreover, the SPAD values were recorded from L11 to L6, and decreased significantly (*p* < 0.01) from L9 to L6 in the MD group after 42 days of Mg starvation treatment (Fig 2B). As Mg is a movable element in plants, it can be transferred from older leaves to younger leaves. The results showed that L9 was the first young leaf to respond to MD among all the leaves. Therefore, we chose L9 for subsequent analysis.

The intercellular CO_2_ concentration was significantly (*p* = 0.02) increased, and the net photosynthetic rate of L9 was significantly (*p* < 0.01) reduced. Moreover, the contents of chlorophyll a, chlorophyll b and carotenoids in different regions of L9 leaves were significantly (*p* = 0.02, 0.02, 0.01) lower under long-term MD treatment in the interior region than in the control group (Fig 2E–2G). Similarly, the same trend was observed in L10 leaves (S2A–S2C Fig).

### RNA sequencing (RNA-seq) and DEG analysis

To further reveal the molecular response mechanism of banana leaves to MD stress, banana L9 under long-term MD for 42 days were subjected to RNA-seq. We constructed four cDNA libraries, namely LM_E, LM_C, LM_I and Control, which represent the edge, center and interior regions of L9 in the MD and control samples. After removing the sequencing adaptor reads and low-quality data, we obtained 41,524,877 reads in the LM_E library, 44,306,550 reads in the LM_C library, 44,682,441 reads in the LM_I library, 36,332,613 reads in the Control library, and 84.64 Gb of RNA-seq data. More than 93.07% of the reads had a quality score of Q30 (Table 1).

**Table 1:** Raw RNA-seq data from the LM-C, LM-E, LM-I and control groups with three replicates.

Sample	Total reads	Clean bases	Q20(%)	Q30(%)	GC (%)	Total mapped	Multiple mapped	Uniquely mapped
LM_C_1	46311602	6915761498	98.01	93.94	50.92	42429478(91.62%)	1164275(2.51%)	41265203(89.1%)
LM_C_2	48060744	7166273315	98.1	94.19	51.43	43952369(91.45%)	1152225(2.4%)	42800144(89.05%)
LM_C_3	51397128	7667685034	98.13	94.24	50.63	46968203(91.38%)	1155248(2.25%)	45812955(89.14%)
LM_E_1	53261110	7965589143	97.98	93.77	51.77	48462220(90.99%)	2508962(4.71%)	45953258(86.28%)
LM_E_2	47497660	7086892299	97.91	93.73	51.89	42771916(90.05%)	1904909(4.01%)	40867007(86.04%)
LM_E_3	42797420	6385557826	97.82	93.51	51.29	39004717(91.14%)	1250350(2.92%)	37754367(88.22%)
LM_I_1	53918060	8041841280	98.08	94.11	51.05	49524657(91.85%)	1014849(1.88%)	48509808(89.97%)
LM_I_2	44475834	6632089788	97.87	93.63	51.26	40599797(91.29%)	1094285(2.46%)	39505512(88.82%)
LM_I_3	51931600	7752182683	98.06	94.07	50.78	47388650(91.25%)	1356648(2.61%)	46032002(88.64%)
Control_1	43947144	6553983269	97.64	93.07	51.19	40147786(91.35%)	840703(1.91%)	39307083(89.44%)
Control_2	42193832	6305807458	97.8	93.44	50.71	38159898(90.44%)	1775168(4.21%)	36384730(86.23%)
Control_3	41266852	6170614481	97.86	93.57	50.29	37520564(90.92%)	1240069(3.01%)	36280495(87.92%)

**Fig 3:**
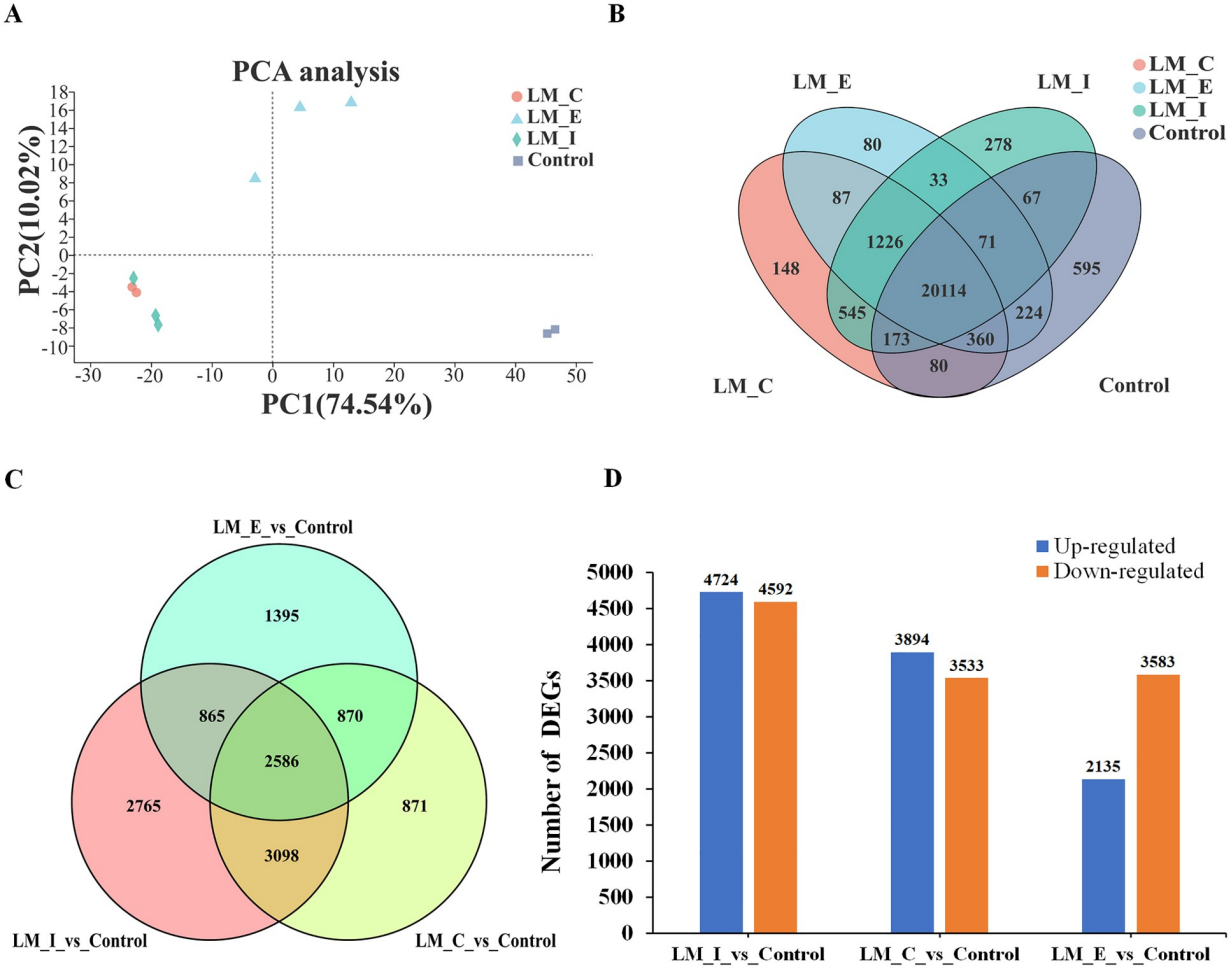
Expression analysis according to treatment (MD and control). **(A)** Principal component analysis (PCA) of gene expression. **(B)** Venn diagram of different samples from the MD and control treatments. **(C)** Venn diagram of the number of shared DEGs between different groups. **(D)** Upregulated and Downregulated DEGs for LM_I vs. Control, LM_C vs. Control and LM_E vs. Control.

Principal component analysis (PCA) was used to evaluate the reliability of samples to explore differences within or between groups. There was a significant difference of 74.54% between LM_E and the control group, while the intragroup difference was 10.02% (Fig 3A). Replicate samples are generally considered reliable and suitable for further analysis. Samples from different groups were clustered together according to the treatment conditions. The control samples were clearly separated from the others, while the LM_I and LM_C samples were clustered together.

A total of 20,114 transcripts were detected in all samples. In total, 80, 148, 278 and 595 transcripts were specifically expressed in the LM_E, LM_C, LM_I and control, respectively (Fig 3B). After using the filtering criteria to determine the significance of the differences in gene expression levels, there were 2,586 coexpressed DEGs in different regions of the L9, while 2,765, 871 and 1,395 DEGs were specifically expressed in LM_I, LM_C and LM_E, respectively (Fig 3C). Compared with the control group, there were 4,724, 3,894 and 2,135 DEGs were upregulated, and 4,592, 3,533 and 3,583 DEGs downregulated in LM_I, LM_C and LM_E, respectively (Fig 3D). Among the 2,586 common DEGs, 1,121, 1,029 and 1,119 DEGs were upregulated, and 1,465, 1,557, 1,467 DEGs were downregulated in LM_I, LM_C and LM_E, respectively (S3 Fig). The results showed that more common DEGs were downregulated than upregulated.

### GO and KEGG enrichment analyses of DEGs

**Fig 4:**
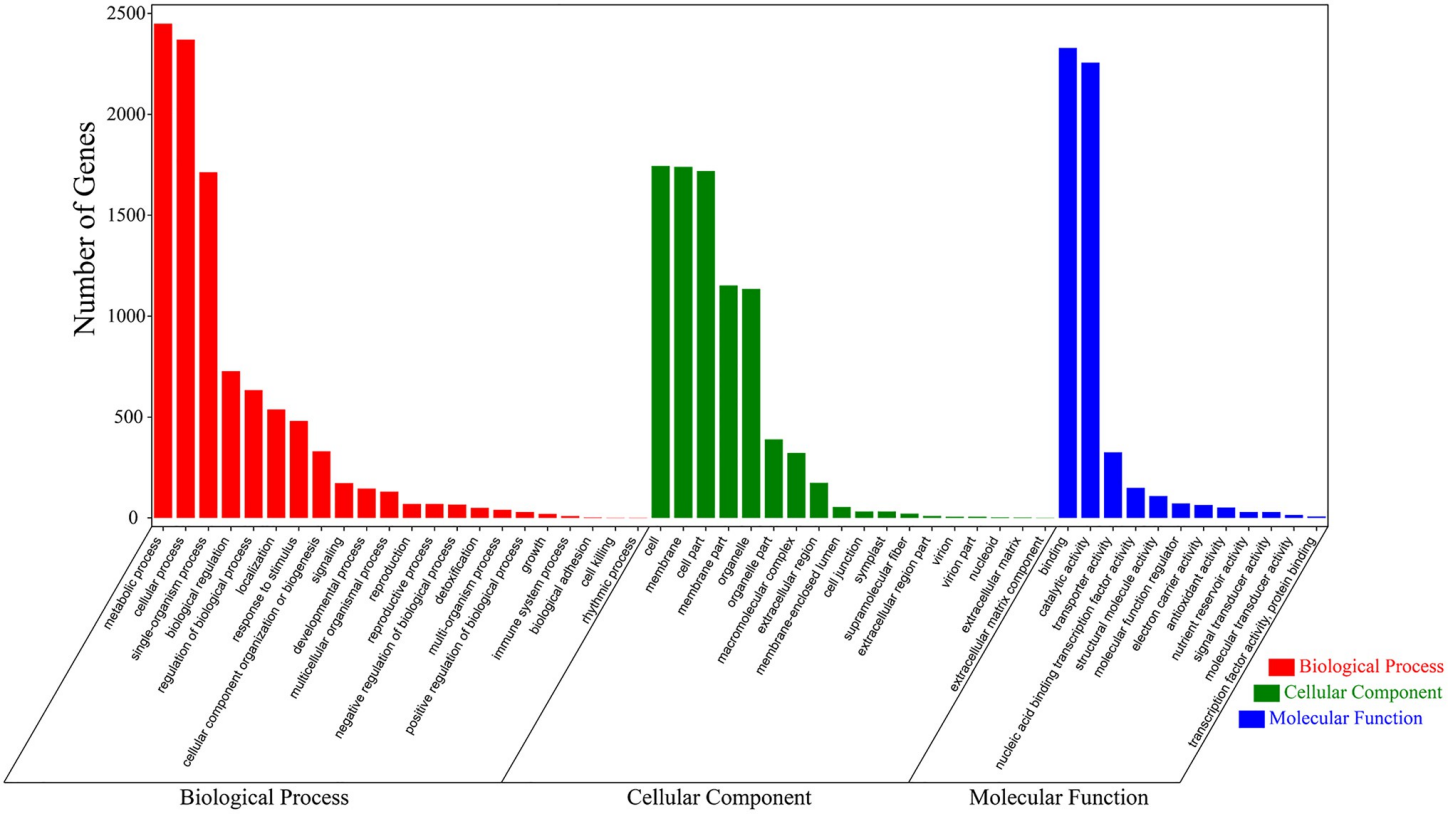
GO classification performed in the transcriptome sequencing dataset.

All DEGs found were classified into the cellular component (CC), molecular function (MF) and biological process (BP) GO categories. For the MF category, the most significant terms included ’binding’, ’catalytic activity’, ’transporter activity’ and ’nucleic acid binding transcription factor activity’. For BP terms, the most significantly overrepresented terms were ’metabolic process’, ’cellular process’, ’single-organism process’, ’biological regulation’ and ’regulation of biological process’. In addition, ’cell’, ’membrane’, ’cell part’, ’membrane part’ and ’organelle’ were significantly overrepresented terms in the CC category (Fig 4).

**Fig 5:**
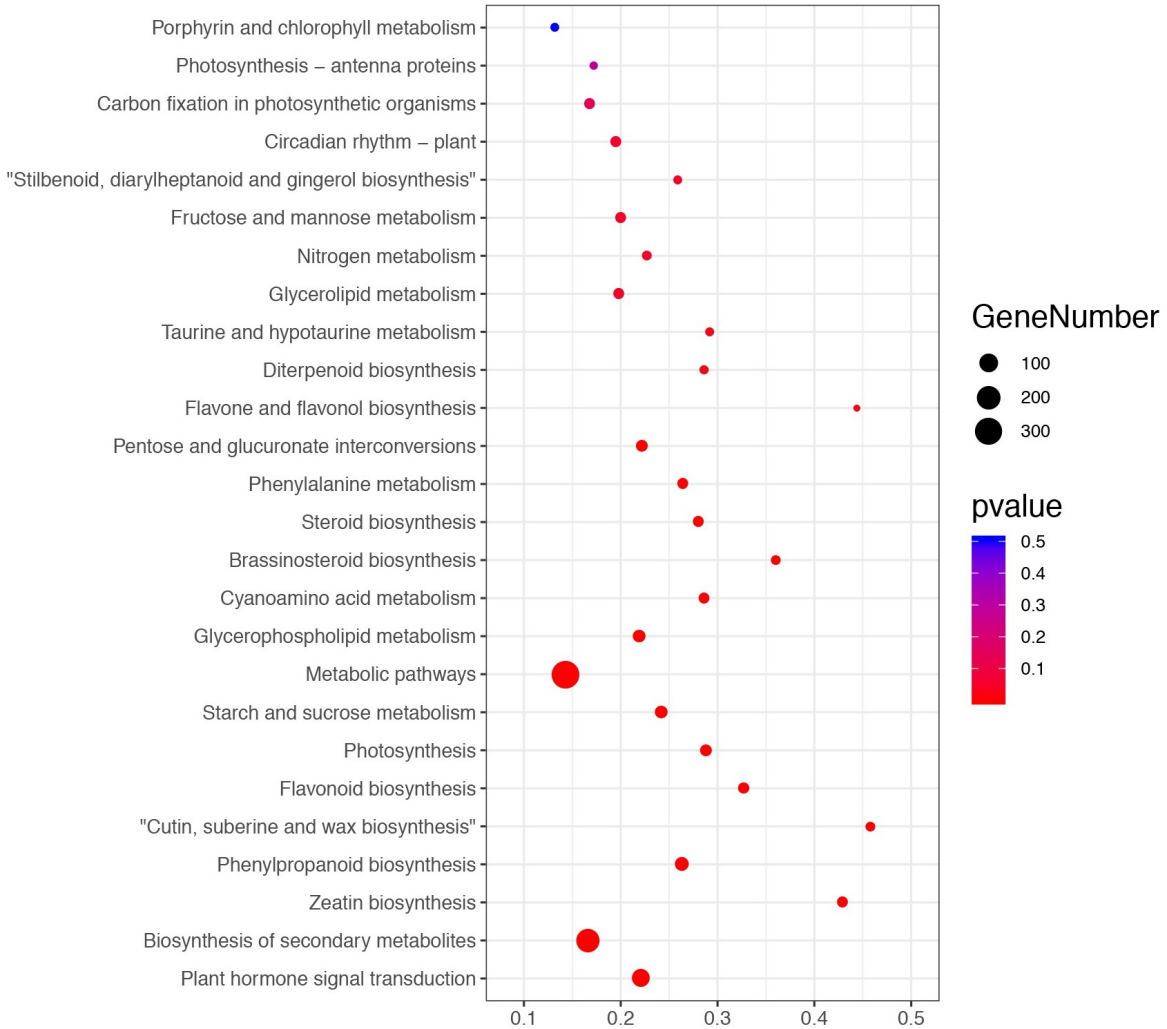
Bubble diagram demonstrating the enrichment of KEGG pathway terms in each module.

In this study, KEGG pathway enrichment analysis of the DEGs revealed 131 pathways that were enriched, including ’carotenoid biosynthesis’, ’carbon fixation in photosynthetic organisms’, ’glycolysis/gluconeogenesis’, ’carbon metabolism’, and ’porphyrin and chlorophyll metabolism’ (Fig 5). According to the KEGG pathway analysis, there were 33 DEGs enriched in the carbon fixation in photosynthetic organisms (ko00710) pathway, including 16 downregulated DEGs and 9 upregulated DEGs. Additionally, 16 DEGs were enriched in the pathway of porphyrin and chlorophyll metabolism pathway (ko00860), of which 10 DEGs were downregulated and 6 DEGs were upregulated.

### DEGs involved in photosynthesis and chlorophyll metabolism

**Fig 6:**
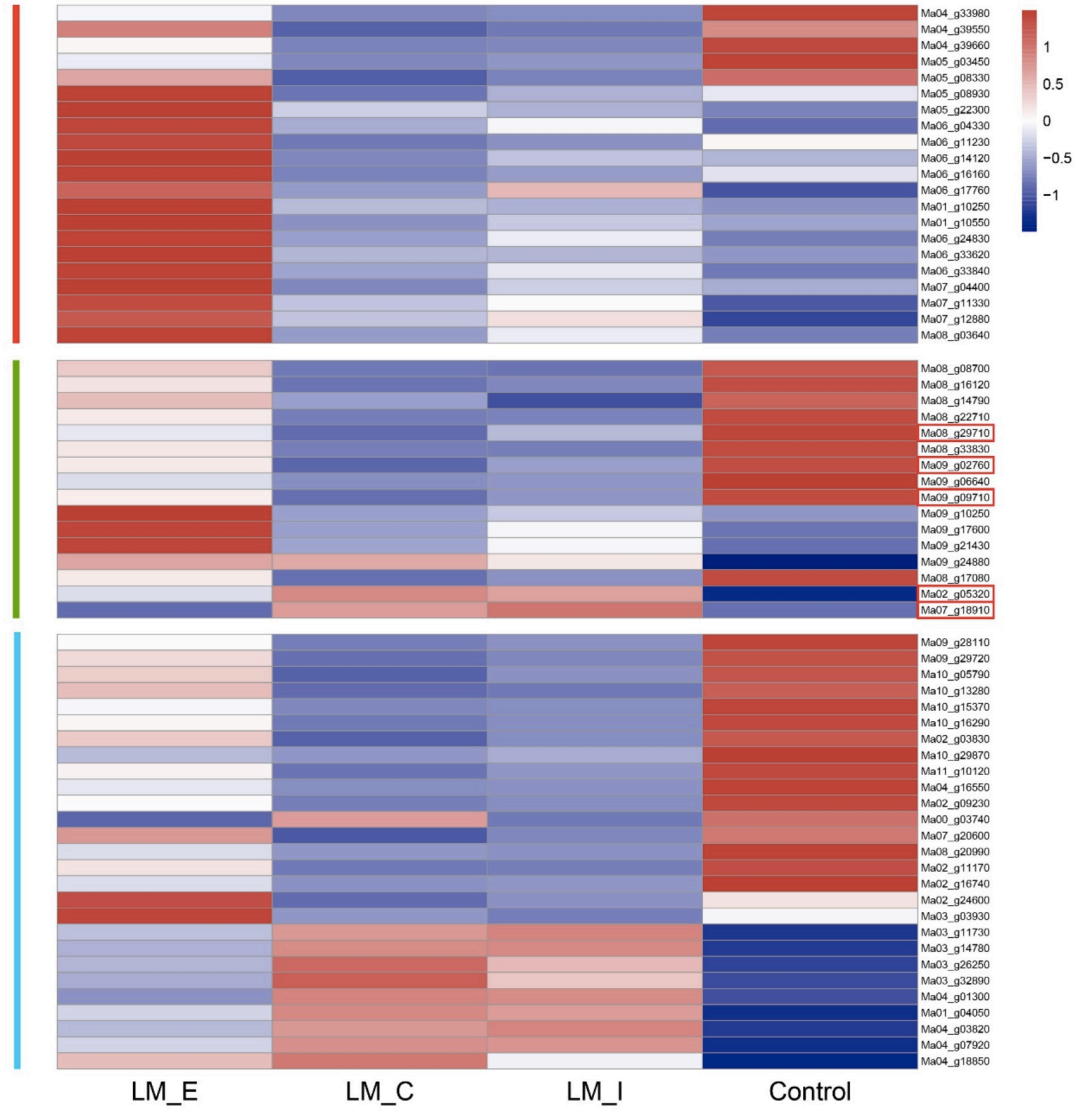
Heatmap generated from the FPKM mean value calculated from three replicates of RNA-seq data. Red represents upregulated genes, and blue represents downregulated genes.

Physiological and transcriptome analyses revealed that MD treatment inhibited the photosynthetic capacity and chlorophyll content in banana leaves. A heatmap of 64 candidate genes differentially expressed between treatments (MD and control) was generated to visualize the expression pattern. A total of 21 genes were associated with photosynthesis-antenna proteins, while 17 genes were upregulated in LM_E (Fig 6).

**Fig 7:**
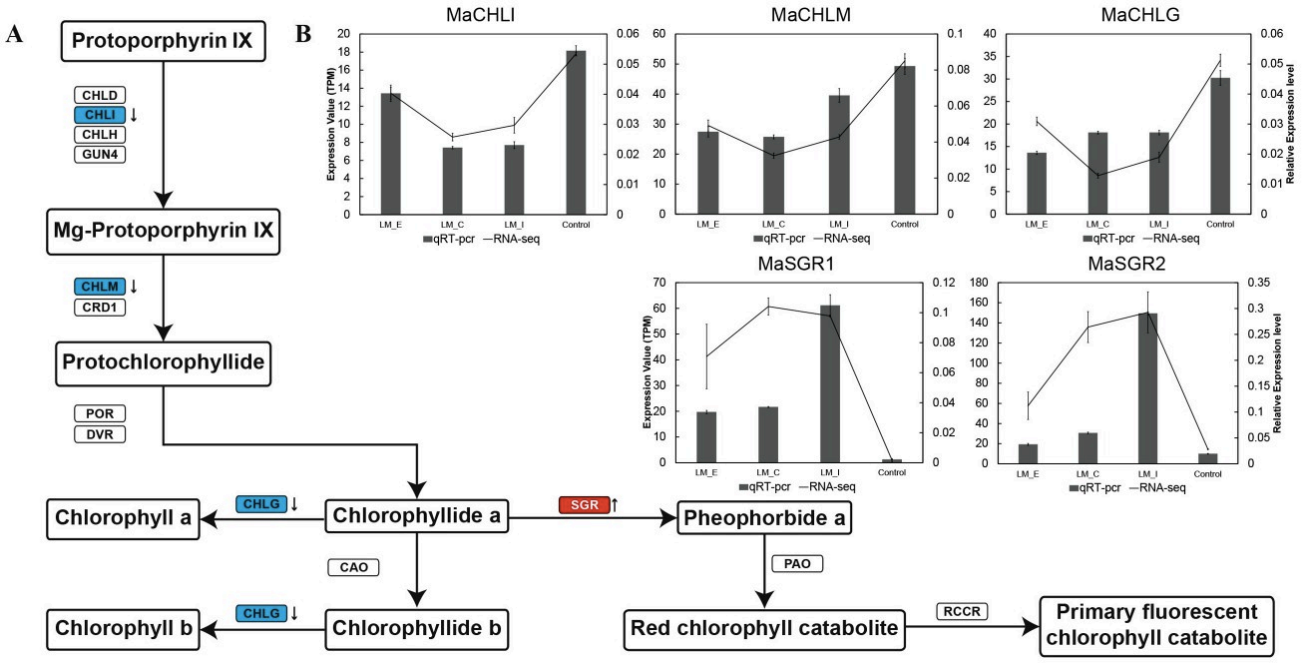
Metabolic network of chlorophyll biosynthesis and degradation in banana leaves. **(A)** DEGs identified by RNA-seq are shown in colored blocks. Blue blocks represent downregulated genes, and red blocks represent upregulated genes. **(B)** qRT-PCR analysis and RNA-seq data of *MaCHLI*, *MaCHLM*, *MaCHLG*, *MaSGR1* and *MaSGR2* expression.

To confirm the accuracy of the RNA-seq data, 5 genes (Ma06_g24000 (*MaCHLI*), Ma09_g24880 (*MaCHLM*), Ma10_g13280 (*MaCHLG*), Ma02_g05320 (*MaSGR1*) and Ma07_g18910 (*MaSGR2*)) related to the chlorophyll metabolism pathway were selected to investigate their expression profiles using qRT-PCR. The verification results of these genes showed similar expression patterns in the RNA-seq data (Fig 7B). Three genes (*MaCHLI*, *MaCHLM* and *MaCHLG*) were downregulated, while 2 genes were upregulated under long-term MD in different banana leaf regions.

## Discussion

Mg^2+^ is transported from roots to aerial tissues by MGR transporters for photosynthesis [37, 38]. Long-term MD triggers dramatic molecular responses in plants including microtubule-based movement, signal transduction, protein phosphorylation and regulation of light harvesting, and the photosynthesis antioxidant system [39, 40]. We characterized the decrease in biomass partitioning and carbohydrate distribution of banana leaves under MD [29]. To gain insight into the molecular response mechanism involved in long-term MD in different regions of banana leaves with the gradual inward extension of yellowing symptoms, RNA-seq analysis was performed to identify key genes and pathways that respond to long-term MD.

For crop plants, the first visual MD symptom is usually interveinal chlorosis in the old leaves owing to the relatively mobile nature of Mg in plants [41]. However, recent studies have shown that Mg remobilization is more vigorous in young mature leaves than in old leaves, which has been verified in sugar beet, *Arabidopsis* and rice [7, 9, 20]. In our experiment, young mature leaves also suffered from marginal chlorosis (Fig 1). These results indicate that Mg transport correlates with leaf vigor. Moreover, MD is known to inhibit the photosynthetic rate and lead to growth retardation and low production [42, 43]. A reduction in the photosynthetic pigment content is always accompanied by photosynthetic inhibition, while the molecular mechanism needs further in-depth study [44]. Here, we showed that the chlorophyll content of banana seedlings under long-term MD significantly decreased, while the photosynthetic pigment contents gradually decreased from the interior regions to the edge (Fig 2). The decrease in the photosynthetic pigment content in response to MD is a common phenomenon [22, 26, 45]. This observation indicates that long-term MD causes a loss of reaction centers connected with the light harvesting complex and photosystem and results in a reduction in the net photosynthetic rate and chlorophyll content.

Photosynthetic processes within chloroplasts require substantial amounts of Mg [46]. MD inhibits photosynthetic capacity and CO_2_ assimilation, which leads to slow metabolism, thus affecting plant growth and development [20, 47, 48]. In *Arabidopsis*, early-term MD altered the expression of circadian clock genes in roots and triggered abscisic acid-responsive genes, whereas long-term MD altered the expression of genes involved in the ethylene biosynthetic pathway, reactive oxygen species detoxification and photoprotection of the photosynthetic apparatus [39, 49]. Long-term MD altered the expression of genes involved in signal transduction, the stress response, carbohydrate and energy metabolism, cell transport, cell wall and cytoskeleton metabolism, and nucleic acid and protein metabolisms in *Citrus reticulata* [50]. MD also regulated genes involved in lignin biosynthesis pathways, regulation of the cell cycle and cell wall metabolism, resulting in lignification, enlargement and cracking of the veins in the lower leaves of *Citrus sinensis* [51]. In our study, the carotenoid biosynthesis, carbon fixation in photosynthetic organisms, glycolysis/gluconeogenesis, carbon metabolism, and porphyrin and chlorophyll metabolism pathways were the most significantly affected according to the KEGG pathway analysis (Fig 5). In contrast to previous studies, we found that the pathway of porphyrin and chlorophyll metabolism pathways were dramatically changed. These results suggested that DEGs induced by long-term MD are involved in the energy and substance metabolism, which affects the growth of banana seedlings. Moreover, different plants responded to MD over varying period time.

According to the RNA-seq data, the majority of altered DEGs were located in the interior regions, whereas the edge had the least number of altered DEGs in L9 under long-term MD (Fig 3). These results suggest that the leaves may suffer from programmed cell death from the edge to the interior regions. In general, the best recognized function of Mg in plants is the formation of chlorophyll pigments, where the red substrate Mg-protoporphyrin IX monomethyl ester is converted to the green product 3,8-divinyl protochlorophyllide a [52]. MgCh catalyzes the first step committed to the synthesis of chlorophyll [53]. MgCh consists of three subunits—CHLH, CHLI, and CHLD—all of which undergo transcriptional and posttranslational modifications in plants and algae [54, 55]. Previous studies showed that the expression of the chlorophyll synthesis genes CHLI, ChlM and CHLG was downregulated under MD [20, 56–58]. Consistent with the KEGG pathway analysis, we found that 3 genes (*MaCHLI*, *MaCHLM* and *MaCHLG*) were simultaneously downregulated in different regions of banana (Fig 6). These results indicate that the chlorophyll biosynthesis capacity is reduced under long-term MD. Moreover, chlorophyll degradation is an important part of nutrient recycling and redistribution during plant stress [59]. *OsSGR* encodes a new chloroplast protein senescence-related gene and regulates chlorophyll degradation in chloroplasts [60]. The qRT-PCR results demonstrated that the expression of the two SGR genes was upregulated, leading to chlorophyll degradation of (Fig 7A). As described, long-term MD treatment inhibited chlorophyll synthesis and promoted chlorophyll degradation, resulting in leaf chlorosis and a decrease in photosynthesis in bananas.

## Conclusion

This study revealed that at the physiological level and transcription levels, MD affected plant chlorosis and senescence processes, namely, the photosynthesis rate, CO_2_ concentration and chlorophyll content, chlorophyll synthesis and degradation-related pathways. The expression of genes related to chlorophyll synthesis and degradation in the three leaf parts—edge, center and interior—was complicated, and involved many plant physiological responses and growth regulatory mechanisms. The results of this study can provide references for the study the functions of Mg in other crops and lay a foundation for the study of the physiological responses and molecular mechanisms of MD in banana.

## Supporting information

S1 Fig Ratio of magnesium ion content to leaf edge (LM_E), center (LM_C) and interior (LM_I) in L7-11. (TIF)

S2 Fig Chlorophyll content in L10 after 42 days of treatment. (**A)** Chla content in different L10 samples. (**B)** Chlb content in different L10 samples. **C**. Car content in different L10 samples.
(TIF)

S3 Fig Histogram showing the common upregulated and downregulated DEGs in LM_I, LM_C and LM_E, respectively. (TIF)

S1 Table List of oligonucleotide sequences used as qRT-PCR primers. (DOCX)
