# Supplementary figures and images for: Chlorophyll decomposition is accelerated in banana leaves after the long-term magnesium deficiency according to transcriptome analysis
Source: PLoS One. 2022 Jun 24;17(6):e0270610. doi: 10.1371/journal.pone.0270610 (PMC9231763; doi:10.1371/journal.pone.0270610)

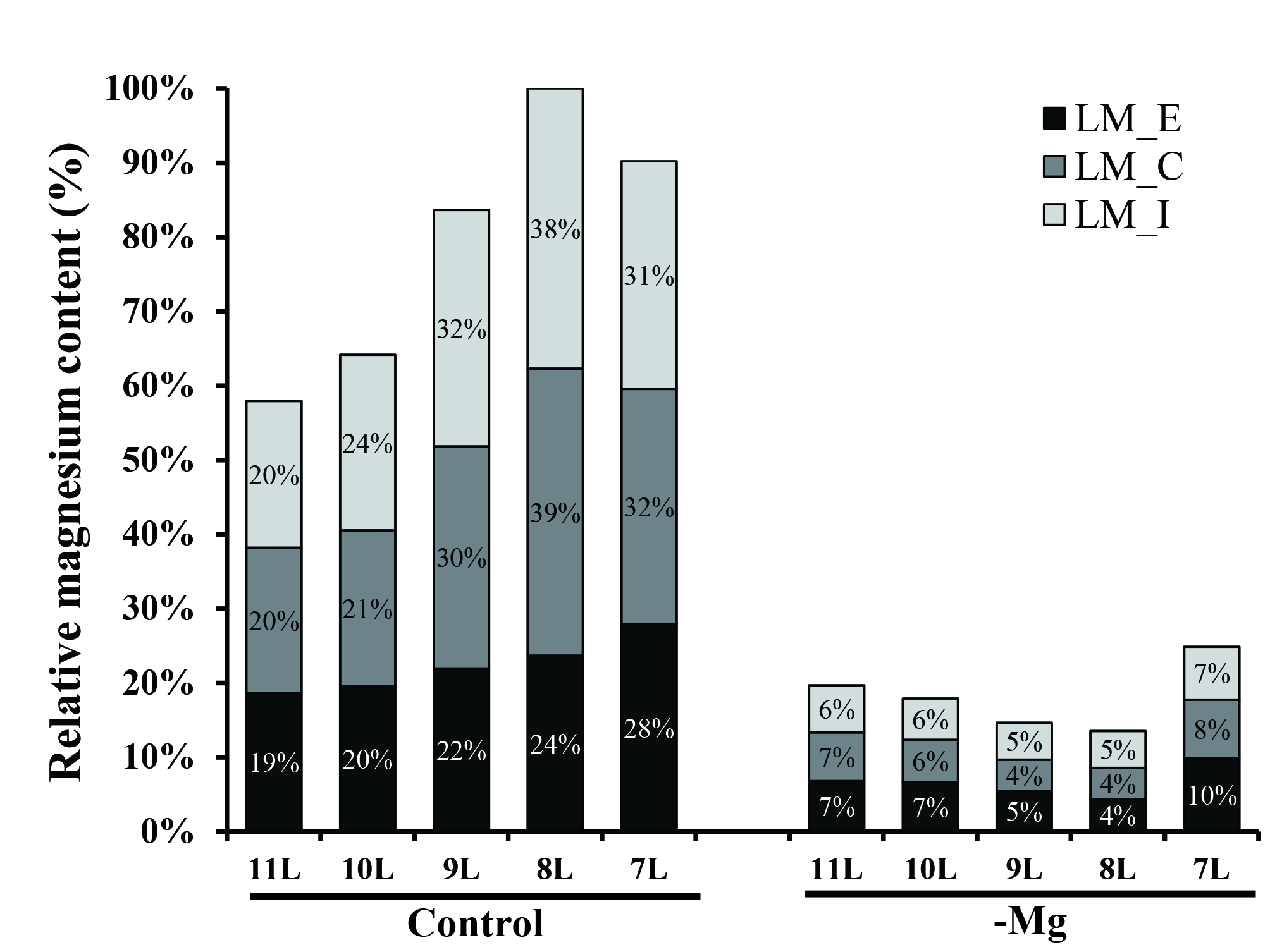

Supplement: S1 Fig — (TIF) [file pone.0270610.s001.tif]

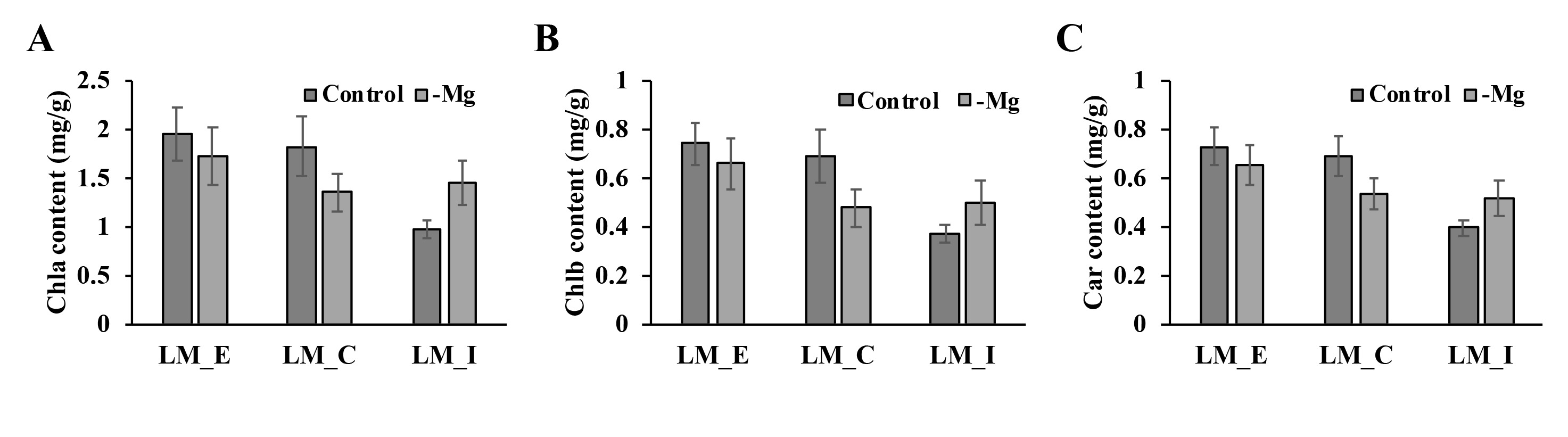

Supplement: S2 Fig — (A) Chla content in different L10 samples. (B) Chlb content in different L10 samples. C. Car content in different L10 samples. (TIF) [file pone.0270610.s002.tif]

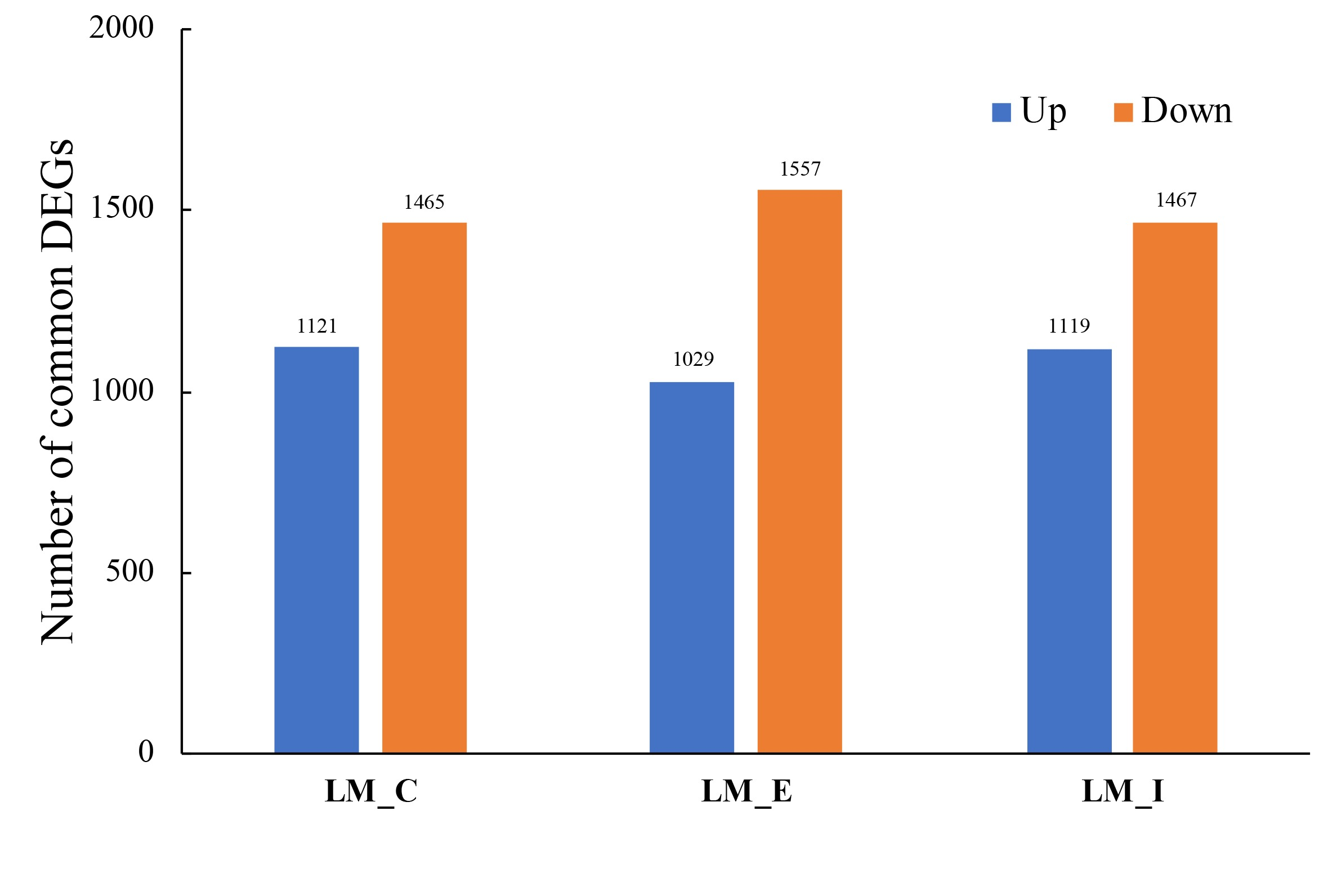

Supplement: S3 Fig — (TIF) [file pone.0270610.s003.tif]
