# Supplementary material for: Chlorophyll decomposition is accelerated in banana leaves after the long-term magnesium deficiency according to transcriptome analysis
Source: PLoS One. 2022 Jun 24;17(6):e0270610. doi: 10.1371/journal.pone.0270610 (PMC9231763; doi:10.1371/journal.pone.0270610)
Supplement: S1 Table — (DOCX) [file pone.0270610.s004.docx]

**Table S1.** List of oligonucleotide sequences used as qRT-PCR primers.

| **Gene name** | Primer Name | Sequence (5'-3') |
| --- | --- | --- |
| Ma08_g29710 | ChlM-F | AGCGGGCTCTGGAGAAGGTC |
|  | ChlM-R | AAGCCTTGTGCCTTTGGTTATC |
| Ma09_g09710 | chlI-F | CACTCTGAGCATTCACTCTAAGAGGT |
|  | chlI-R | ACAAGAACAAATACAAAACTGCAAGG |
| Ma09_g02760 | chlG-F | TGATGTCAAATACCAGGCTAGTGC |
|  | chlG-R | TACTGTCTGAGGATGCCGGGAT |
| HQ853246 | RPS2-F | TAGGGATTCCGACGATTTGTTT |
|  | RPS2-R | TAGCGTCATCATTGGCTGGGA |
| Ma02_g05320 | SGR1-F | CCATCCTTAATTCTTTGAATTGATGA |
|  | SGR1-R | CATCTTTTTACAAACCATTGATACACCT |
| Ma07_g18910 | SGR2-F | CTGGCCACACGATCTCGAAG |
|  | SGR2-R | AATCTGTGTTGTATGATTAGACTGCATC |
